# Bedside lung ultrasound for the diagnosis of pneumonia in children presenting to an emergency department in a resource-limited setting

**DOI:** 10.1186/s12245-022-00474-w

**Published:** 2023-01-09

**Authors:** Yogendra Amatya, Frances M. Russell, Suraj Rijal, Sunil Adhikari, Benjamin Nti, Darlene R. House

**Affiliations:** 1 Department of General Practice and Emergency Medicine, Patan Academy of Health Sciences, Kathmandu, Nepal; 2 Department of Emergency Medicine, Indiana University School of Medicine, Indianapolis, IN USA; 3 Departments of Global Health and Emergency Medicine, Icahn School of Medicine at Mount Sinai, New York, NY USA

**Keywords:** Lung ultrasound, Pneumonia, Diagnosis, Developing countries, Pediatric

## Abstract

**Background:**

Lung ultrasound (LUS) is an effective tool for diagnosing pneumonia; however, this has not been well studied in resource-limited settings where pneumonia is the leading cause of death in children under 5 years of age.

**Objective:**

The objective of this study was to evaluate the diagnostic accuracy of bedside LUS for diagnosis of pneumonia in children presenting to an emergency department (ED) in a resource-limited setting.

**Methods:**

This was a prospective cross-sectional study of children presenting to an ED with respiratory complaints conducted in Nepal. We included all children under 5 years of age with cough, fever, or difficulty breathing who received a chest radiograph. A bedside LUS was performed and interpreted by the treating clinician on all children prior to chest radiograph. The criterion standard was radiographic pneumonia, diagnosed by a panel of radiologists using the Chest Radiography in Epidemiological Studies methodology. The primary outcome was sensitivity and specificity of LUS for the diagnosis of pneumonia. All LUS images were later reviewed and interpreted by a blinded expert sonographer.

**Results:**

Three hundred and sixty-six children were enrolled in the study. The median age was 16.5 months (IQR 22) and 57.3% were male. Eighty-four patients (23%) were diagnosed with pneumonia by chest X-ray. Sensitivity, specificity, positive and negative likelihood ratios for clinician’s LUS interpretation was 89.3% (95% CI 81–95), 86.1% (95%CI 82–90), 6.4, and 0.12 respectively. LUS demonstrated good diagnostic accuracy for pneumonia with an area under the curve of 0.88 (95% CI 0.83–0.92). Interrater agreement between clinician and expert ultrasound interpretation was excellent (*k* = 0.85).

**Conclusion:**

Bedside LUS when used by ED clinicians had good accuracy for diagnosis of pneumonia in children in a resource-limited setting.

## Introduction

Pneumonia is the leading cause of death in children under 5 years of age worldwide [1]. Timely diagnosis and treatment is critical to reduce morbidity and mortality [2]. In resource-limited settings, clinicians primarily diagnose pneumonia in children based on the World Health Organization criteria; however, multiple studies have shown these criteria lack sensitivity and specificity, leading to misdiagnosis and over treatment [3–5]. Chest radiographs can be used to confirm suspected pneumonia; however, in resource-limited settings its use is limited as remote facilities often lack radiographic imaging capabilities.

Lung ultrasound is an effective tool for diagnosis of pneumonia in both adults and children [6–10]. Ultrasound is easily portable, readily available at many facilities, and not associated with radiation. Despite the fact that ultrasound is highly sensitive and specific for pneumonia, few studies have evaluated the accuracy of lung ultrasound for pneumonia in pediatric patients in a resource-limited setting [6, 7, 11–14].

The objective of this study was to evaluate the diagnostic accuracy of bedside LUS for the diagnosis of pneumonia in children presenting to an emergency department (ED) in a resource-limited setting.

## Methodology

### Study design

This was a prospective observational cross-sectional study of pediatric patients presenting with fever or respiratory complaints to the ED at Patan Hospital in Lalitpur, Nepal. The study was performed from May 2018 to March 2020 when the study was stopped due to the COVID-19 pandemic. Additionally, the study was halted for ten months during that time period due to ultrasound maintenance. Ethical approval was obtained from the Patan Academy of Health Sciences Institutional Review Committee.

### Study setting and population

Patan Hospital is a large urban hospital with a 35-bed ED. The ED has an annual volume of approximately 48,000 patients, including approximately 8000 pediatric visits. The overall admission rate is 20%.

Patients were included in the study if they were under the age of 5 years, presented to the ED with a chief complaint of fever or had a respiratory complaint (i.e., cough, difficulty breathing), if there was concern by the treating clinician for pneumonia, and a chest X-ray was ordered. Patients were excluded if a chest radiograph was not performed.

### Study protocol

Written informed consent was obtained from parents of patients meeting the inclusion criteria.

A bedside lung ultrasound (LUS) was then performed on all children prior to chest X-ray being performed. Ultrasound was performed by 22 different treating clinicians, including medical officers, residents, emergency medicine (EM) fellows, and EM faculty. Clinicians performing LUS were not blinded to clinical information, but were blinded to chest radiograph images and results. A Sonosite M Turbo (Fujifilm Sonosite, Inc.) ultrasound machine with a curvilinear probe was used. In accordance with previous literature, the ultrasound examination included ten views: two anterior views and two lateral views (one including the costophrenic angle), and one posterior view in each hemithorax [6, 15]. Ultrasound findings and interpretation were recorded on a standardized data collection form. An ultrasound diagnosis of pneumonia was defined as the presence of unilateral focal B-lines or subpleural consolidation.

All patients had a single posterior-anterior chest X-ray as a part of their standard evaluation. Two-view chest X-ray is not standard in this setting, as patients then have to pay for two X-rays. The WHO Chest Radiography in Epidemiological Studies (CRES) methodology was used to standardize interpretation of chest X-rays and used to define radiographic pneumonia. Two Nepali general radiologists trained in WHO CRES methodology, who were blinded to the clinical presentation and each other’s interpretations, independently read the chest X-rays. Any discordant reads were adjudicated by a board-certified pediatric radiologist who also completed the WHO CRES methodology training.

### Statistical analysis

An estimated sample size of 453 patients was calculated for the study based on an expected sensitivity of 92% for LUS with a 95% confidence level, precision of 0.05, and prevalence of 0.25 [7].

Diagnostic test characteristics for lung ultrasound were calculated, including sensitivity, specificity, and positive and negative predictive values, and likelihood ratios. A receiver operator curve analysis was performed to evaluate the diagnostic accuracy of lung ultrasound for the diagnosis of pneumonia.

Kappa analysis was done to determine the inter-rater reliability between the clinician’s lung ultrasound interpretation and an expert sonographer’s interpretation. The expert sonographer was blinded to the clinician’s interpretation. All analyses were completed using IBM SPSS Statistics version 26.0 (Armonk, NY).

## Results

Three hundred and sixty-six children were enrolled in the study; however, one child had to be excluded from the study as the chest X-ray was uninterpretable based on the CRES methodology. The median age was 16.5 months (IQR 22) with 57.3% male (Table 1).

**Table 1:** Patient demographics

	Total *n* (%) *N* = 365
Age (months)	
< 2	19 (5.2)
2–11	111 (30.4)
12–23	89 (24.4)
24–35	65 (17.8)
36–47	43 (11.7)
48–60	38 (10.4)
Female	156 (42.7)

Eighty-four patients (23%) were diagnosed with pneumonia by chest X-ray. For the diagnosis of pneumonia, the clinician’s LUS interpretation had a sensitivity of 89.3% (95% CI 81–95), specificity of 86.1% (95%CI 82–90), positive likelihood ratio of 6.4, and negative likelihood ratio of 0.12 (Table 2).

**Table 2:** Lung ultrasound compared to chest X-ray for pneumonia

	CXR positive (*n* = 84)	CXR negative (*n* = 281)
Lung ultrasound		
Positive	75	39
Negative	9	242

**Fig. 1:**
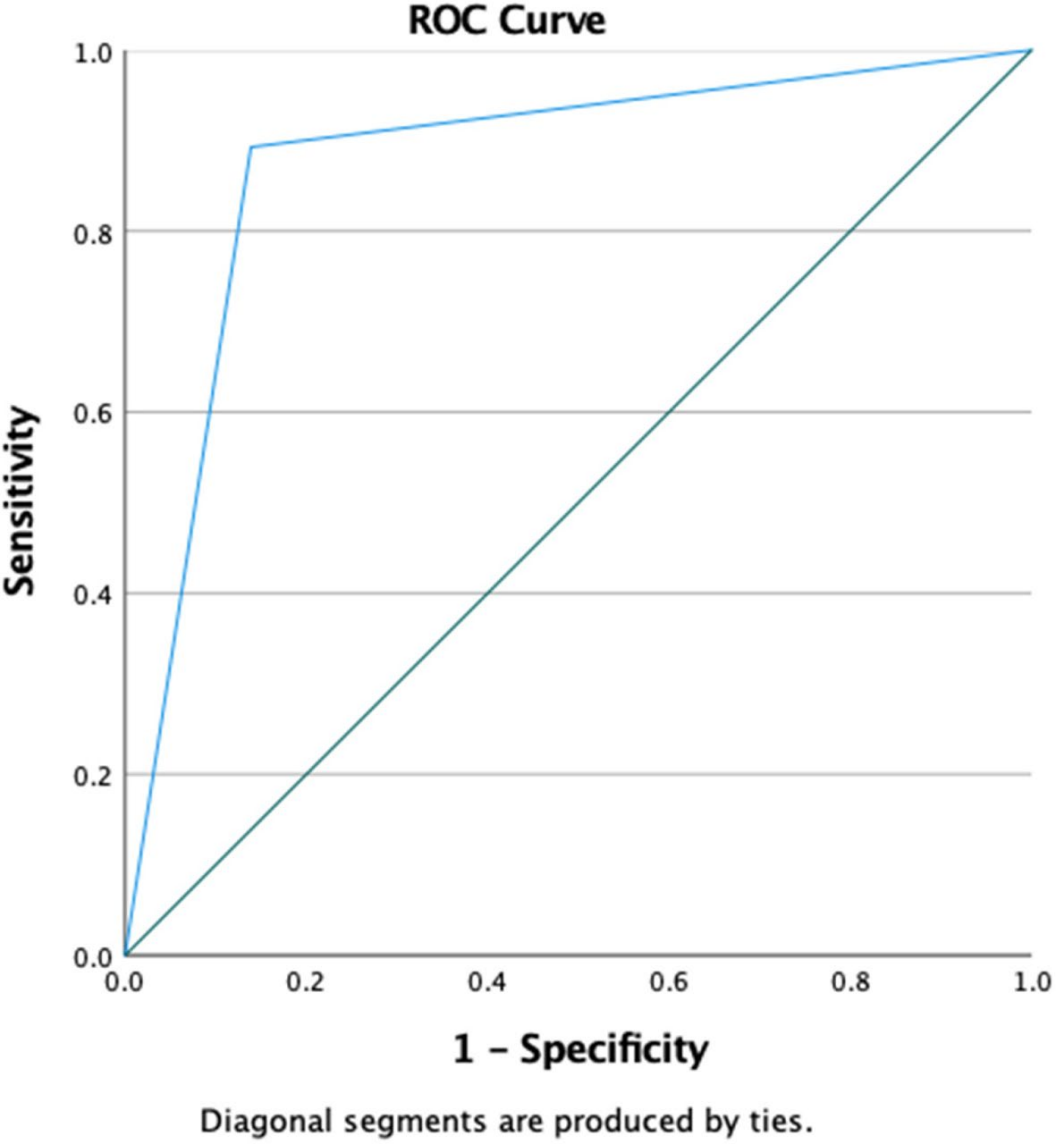
ROC analysis for LUS for pneumonia in children

LUS demonstrated good diagnostic accuracy for pneumonia with an area under the curve of 0.88 (95% CI 0.83–0.92) (Fig. 1). LUS were performed and submitted by 22 different clinicians. Agreement between the clinician and expert ultrasound interpretation was excellent (*k* = 0.85). The inter-rater reliability between expert chest X-ray reads was good (*k* = 0.61).

## Discussion

This study is the first study in Nepal to demonstrate accuracy of lung ultrasound for diagnosis of pneumonia in children presenting to an emergency department. It is one of only a few studies performed in resource-limited settings. In this study, we found that LUS demonstrated a sensitivity of 89% and specificity of 86% for the diagnosis of pneumonia in an ED pediatric patient population.

Our study found similar results to studies performed in both resource-rich and resource-limited settings. Two recent meta-analyses evaluating lung ultrasound for the diagnosis of pneumonia in children found LUS to have sensitivity of 94 to 96% and a specificity of 93% [9, 13]. These meta-analyses primarily included studies performed in resource-rich settings and may not be generalizable to resource-limited settings. When looking at data from a resource-limited setting, one study from Peru performed in the emergency, inpatient, and outpatient settings demonstrated good diagnostic accuracy for LUS with a sensitivity of 88 to 92% and specificity of 100% [7]. Yadav et al. also evaluated LUS compared to chest radiographs in children hospitalized with pneumonia in India, demonstrating a sensitivity of 98%; however, they found a specificity of only 65% [16].

Our study focused on emergency department patients and found similar sensitivity to previous studies. However, we anticipated a higher specificity, but only found a specificity of 86%. Overall, there were 39 false-positive results. The lower specificity we found in this study could be due to the standard used [7, 9]. Chest X-ray has been used as the criterion standard in previous diagnostic studies on pneumonia, but lacks sensitivity [9, 17]. Because of this, it is possible that chest X-ray missed pneumonias that ultrasound was able to detect [8, 18].

In our study, we had nine false negative ultrasounds. All of these ultrasounds were interpreted as normal by both the clinician and blinded expert sonographer. These missed pneumonias may be due to operator error in placement of the probe or lack of complete coverage of the chest with the scanning protocol. Also, pneumonia located in the middle of the lung parenchyma that does not extend to the pleura can be missed by ultrasound [19]. Half of these children with missed pneumonia on lung ultrasound had already been on antibiotic therapy from a pharmacy prior to ED evaluation. Resolution of abnormalities on chest X-rays may take weeks after treatment, potentially longer than ultrasound which may account for these diagnostic discrepancies between ultrasound and X-ray [20].

In our study, LUS was performed by 22 different physicians with varied levels of medical and ultrasound training. Ultrasound examinations were independently interpreted by two expert faculty with excellent agreement compared to clinicians, better than the agreement between independent radiologist reads for chest X-rays. While ultrasound is an operator-dependent skill, this study demonstrates that it can be utilized by many providers in the care of patients presenting to the emergency department. Further research should focus on the implementation of lung ultrasound in the evaluation of children with suspected pneumonia, and specifically addressing ways to help improve diagnosis, treatment, and outcomes in resource-limited settings.

## Limitations

Our study has several limitations. The primary limitation is the use of chest radiograph as the criterion standard, which has limited sensitivity for pneumonia [17, 18, 21]. However, chest radiograph is often the standard used in the clinical setting for diagnosis and recommended as the imaging modality of choice for pneumonia in children [22]. We did not compare the findings of ultrasound with computed tomography (CT). While CT is the gold standard for diagnosing pneumonia and provides more accurate results, it is not recommended for use in children due to radiation exposure [23]. Due to the lack of funding and infrastructure, we were unable to track all eligible patients so our sample is a convenience sample and may be subject to selection bias. We also had limitations in data collection due to equipment failure; however, this represents the reality in resource-limited settings and highlights the need for affordable and locally-repairable equipment. Finally, we had to end the study early due to the COVID-19 pandemic. Despite having wider confidence intervals, the results are similar to previous studies.

## Conclusion

LUS performed by ED providers had good accuracy for the diagnosis of pneumonia in children in a resource-limited setting.

## Data Availability

The datasets used and/or analyzed during the current study are available from the corresponding author on reasonable request.

## Abbreviations

LUS: Lung ultrasound
ED: Emergency department
WHO: World Health Organization
CRES: Chest Radiography in Epidemiological Studies

## References

[1] World Health Organization. Children: improving survival and well-being [updated September 2020]. Available from: https://www.who.int/news-room/fact-sheets/detail/children-reducing-mortality. Accessed Nov 2021.

[2] Mortensen EM, Restrepo MI, Anzueto A, Pugh JA (2006). Antibiotic therapy and 48-hour mortality for patients with pneumonia. Am J Med.

[3] World Health Organization. Revised WHO classification and treatment of childhood pneumonia at health facilities: Evidence Summaries. Geneva: Switzerland; 2014. p. 1-6. Available from: https://www.who.int/publications/i/item/9789241507813. Accessed Nov 2021. 25535631

[4] House DR, Rijal S, Adhikari S, Cooper ML, Hohl CM (2020). Prospective evaluation of World Health Organization guidelines for diagnosis of pneumonia in children presenting to an emergency department in a resource-limited setting. Paediatr Int Child Health.

[5] Chavez MA, Naithani N, Gilman RH, Tielsch JM, Khatry S, Ellington LE (2015). Agreement between the world health organization algorithm and lung consolidation identified using point-of-care ultrasound for the diagnosis of childhood pneumonia by general practitioners. Lung..

[6] Cortellaro F, Colombo S, Coen D, Duca PG (2012). Lung ultrasound is an accurate diagnostic tool for the diagnosis of pneumonia in the emergency department. Emerg Med J.

[7] Ellington LE, Gilman RH, Chavez MA, Pervaiz F, Marin-Concha J, Compen-Chang P (2017). Lung ultrasound as a diagnostic tool for radiographically-confirmed pneumonia in low resource settings. Respir Med.

[8] Shah VP, Tunik MG, Tsung JW (2013). Prospective evaluation of point-of-care ultrasonography for the diagnosis of pneumonia in children and young adults. JAMA Pediatr.

[9] Orso D, Ban A, Guglielmo N (2018). Lung ultrasound in diagnosing pneumonia in childhood: a systematic review and meta-analysis. J Ultrasound.

[10] Orso D, Guglielmo N, Copetti R (2018). Lung ultrasound in diagnosing pneumonia in the emergency department: a systematic review and meta-analysis. Eur J Emerg Med.

[11] Bourcier JE, Paquet J, Seinger M, Gallard E, Redonnet JP, Cheddadi F (2014). Performance comparison of lung ultrasound and chest x-ray for the diagnosis of pneumonia in the ED. Am J Emerg Med.

[12] Liu XL, Lian R, Tao YK, Gu CD, Zhang GQ (2015). Lung ultrasonography: an effective way to diagnose community-acquired pneumonia. Emerg Med J.

[13] Pereda MA, Chavez MA, Hooper-Miele CC, Gilman RH, Steinhoff MC, Ellington LE (2015). Lung ultrasound for the diagnosis of pneumonia in children: a meta-analysis. Pediatrics..

[14] Xin H, Li J, Hu HY. Is lung ultrasound useful for diagnosing pneumonia in children?: A meta-analysis and systematic review. Ultrasound Q. 2018;34(1):3-10. 10.1097/RUQ.0000000000000330 29112644

[15] Lichtenstein DA, Lascols N, Meziere G, Gepner A (2004). Ultrasound diagnosis of alveolar consolidation in the critically ill. Intensive Care Med.

[16] Yadav KK, Awasthi S, Parihar A (2017). Lung ultrasound is comparable with chest roentgenogram for diagnosis of community-acquired pneumonia in hospitalised children. Indian J Pediatr.

[17] Hagaman JT, Rouan GW, Shipley RT, Panos RJ (2009). Admission chest radiograph lacks sensitivity in the diagnosis of community-acquired pneumonia. Am J Med Sci.

[18] Self WH, Courtney DM, McNaughton CD, Wunderink RG, Kline JA (2013). High discordance of chest x-ray and computed tomography for detection of pulmonary opacities in ED patients: implications for diagnosing pneumonia. Am J Emerg Med.

[19] Reissig A, Copetti R, Mathis G, Mempel C, Schuler A, Zechner P (2012). Lung ultrasound in the diagnosis and follow-up of community-acquired pneumonia: a prospective, multicenter, diagnostic accuracy study. Chest..

[20] Bruns AH, Oosterheert JJ, Prokop M, Lammers JW, Hak E, Hoepelman AI (2007). Patterns of resolution of chest radiograph abnormalities in adults hospitalized with severe community-acquired pneumonia. Clin Infect Dis.

[21] Karimi E (2019). Comparing Sensitivity of Ultrasonography and Plain Chest Radiography in Detection of Pneumonia; a Diagnostic Value Study. Arch Acad Emerg Med.

[22] Bradley JS, Byington CL, Shah SS, Alverson B, Carter ER, Harrison C (2011). Executive summary: the management of community-acquired pneumonia in infants and children older than 3 months of age: clinical practice guidelines by the Pediatric Infectious Diseases Society and the Infectious Diseases Society of America. Clin Infect Dis.

[23] O'Grady KF, Torzillo PJ, Frawley K, Chang AB (2014). The radiological diagnosis of pneumonia in children. Pneumonia (Nathan).

